# Supplementary material for: DNA sequence encodes the position of DNA supercoils
Source: eLife. 2018 Dec 7;7:e36557. doi: 10.7554/eLife.36557 (PMC6301789; doi:10.7554/eLife.36557)
Supplement: Supplementary file 1. [file elife-36557-supp1.docx]

**Supplementary File 1**

Parameters used for calculating the free path of DNA. Direction, wedge, and twist are from reference 28 (Balasubramanian et al 2009) and Tilt-Tilt and Roll-Roll covariance are from reference 29 (Lankaš et al 2003).

| **Dinucleotide** | **Direction (ϕ_B_, degrees)** | **Wedge (θ, degrees)** | **Twist (degrees)** | **Tilt-Tilt covariance** | **Roll-Roll covariance** |
| --- | --- | --- | --- | --- | --- |
| AA | -153.938 | 7.197 | 35.606 | 0.686 | 1.135 |
| AC | 142.942 | 1.100 | 34.386 | 0.649 | 0.999 |
| AG | 1.999 | 8.397 | 27.689 | 0.719 | 1.175 |
| AT | 0.000 | 2.599 | 31.487 | 0.660 | 0.981 |
| CA | -63.974 | 3.499 | 34.486 | 0.970 | 1.450 |
| CC | -56.977 | 2.099 | 33.656 | 0.644 | 1.107 |
| CG | 0.000 | 6.697 | 29.788 | 0.960 | 1.744 |
| CT | -1.999 | 8.397 | 27.689 | 0.719 | 1.175 |
| GA | 119.952 | 5.298 | 36.885 | 0.681 | 1.264 |
| GC | 179.927 | 4.998 | 39.984 | 0.674 | 0.970 |
| GG | 56.977 | 2.099 | 33.656 | 0.644 | 1.107 |
| GT | -142.942 | 1.100 | 34.386 | 0.649 | 0.999 |
| TA | 0.000 | 0.900 | 35.986 | 1.089 | 1.962 |
| TC | -119.952 | 5.298 | 36.885 | 0.681 | 1.264 |
| TG | 63.974 | 3.499 | 34.486 | 0.970 | 1.450 |
| TT | 153.938 | 7.197 | 35.606 | 0.686 | 1.135 |
