# Supplementary material for: DNA sequence encodes the position of DNA supercoils
Source: eLife. 2018 Dec 7;7:e36557. doi: 10.7554/eLife.36557 (PMC6301789; doi:10.7554/eLife.36557)
Supplement: Supplementary file 2. [file elife-36557-supp2.docx]

**Supplementary File 2**

| **Sequence of Interest** | |
| --- | --- |
| SeqA (Fig. 2b) | CGCTATTCTTCGGTTAACGAAGTCTGTAATTGATTTGTTAAGTTTTTCTTTTGAATATACGCATTAATTTTAGATTGAATTTGCTGAAGCTCTTCTTGATCAGCTTCTCGAATGGTTTGCTCCTTTTGTTCGCTCGTTAACGATTGTGAAGAAGCAGGAGACTGATTAGGGGCTGTCTCCACTTCATTTGTTTTTTTCGTACTTTTAGCTGCAGGGGTATTTGAAGGCTGAACCGCTTCTTCAGATTCCTGCAGAGATTGAAATTCCATCACGCCCGTTCCTCCAACGAACGCTTCATTAAATGCGCGAGAAAGGTTCTTAAATTTCTGCGCATCCACTGAGCTCATTGCAAACAATACAATGAATAAAGCAAGTAAAAGTGTAAGCAAATCTGAGTAAGGAAGCAGCCAGCTTTCGTCAACATGGTCCTCTTCATGCTTTCGTTTTCTGCGTCTACTCATTTATGCCCACTTCACTTTCTTGAAGAAGCTTTTTACGTTCCGCTGTTGGCAAATAAGAAGCCAGCTTTTGCTCAATTACTTTTGGTGTTTCTCCTTCTAAAAGTGAAAGCACTCCTTCGATCATCATATACTTTACCTTTACTTCATGTTTCGATTTACGCTTTAGTTTATTTGCAAACGGATGCCATAGTACATACCCAGTAAAAATACCAAGAAGCGTAGCAACAAACGCCGCGCTGATCGCATGTCCTAGCGTATCTGTATCTTCCATGTTCCCAAGCGCAGCAATTAACCCTATAACAGCTCCAAGTACACCCAGAGTTGGAGCATATGTACCTGCTAAAGCGAAAATACTTGCACCCGTTTGATGTCTTTCTTCCATAGCATCAATTTCTTCAGACAACACGTCTCGTATATAATCCGCACTTTGACCATCAATAGCTAAATTCAAACCATTTTTTAAGAAAGGGTCATCTACATCAATAATTTGGGCTTCAAGTGATAGTAACCCTTCTTTTCGAACAACTTGTCCCCATTCAGAAAACGCGT |
| SeqB (Fig. 2b) | CGCTTACTCGTGCACCCAACTGATCTTCAGCATCTTTTACTTTCACCAGCGTTTCTGGGTGAGCAAAAACAGGAAGGCAAAATGCCGCAAAAAAGGGAATAAGGGCGACACGGAAATGTTGAATACTCATACTCTTCCTTTTTCAATATTATTGAAGCATTTATCAGGGTTATTGTCTCATGAGCGGATACATATTTGAATGTATTTAGAAAAATAAACAAATAGGGGTTCCGCGCACATTTCCCCGAAAAGTGCCACCTAAATTGTAAGCGTTAATATTTTGTTAAAATTCGCGTTAAATTTTTGTTAAATCAGCTCATTTTTTAACCAATAGGCCGAAATCGGCAAAATCCCTTATAAATCAAAAGAATAGACCGAGATAGGGTTGAGTGTTGTTCCAGTTTGGAACAAGAGTCCACTATTAAAGAACGTGGACTCCAACGTCAAAGGGCGAAAAACCGTCTATCAGGGCGATGGCCCACTACGTGAACCATCACCCTAATCAAGTGGCGT |
| SeqC (Fig. 2b) | CGCTTTTGGTAGCTCTTGATCCGGCAAACAAACCACCGCTGGTAGCGGTGGTTTTTTTGTTTGCAAGCAGCAGATTACGCGCAGAAAAAAAGGATCTCAAGAAGATCCTTTGATCTTTTCTACGGGGTCTGACGCTCAGTGGAACGAAAACTCACGTTAAGGGATTTTGGTCATGAGATTATCAAAAAGGATCTTCACCTAGATCCTTTTAAATTAAAAATGAAGTTTTAAATCAATCTAAAGTATATATGAGTAAACTTGGTCTGACAGTTACCAATGCTTAATCAGTGAGGCACCTATCTCAGCGATCTGTCTATTTCGTTCATCCATAGTTGCCTGACTCCCCGTCGGCGT |
| seqCopy (Fig. 2d) | CGCTCCGCTACGAAATGCGCGTATGGGGATGGGGGCCGGGTGAGGAAAGCTGGCTGATTGACCGGCAGATTATTATGGGCCGCCACGACGATGAACAGACGCTGCTGCGTGTGGATGAGGCCATCAATAAAACCTATACCCGCCGGAATGGTGCAGAAATGTCGATATCCCGTATCTGCTGGGATACTGGCGGGATTGACCCGACCATTGTGTATGAACGCTCGAAAAAACATGGGCTGTTCCGGGTGATCCCCATTAAAGGGGCATCCGTCTACGGAAAGCCGGTGGCCAGCATGCCACGTAAGCGAAACAAAAACGGGGTTTACCTTACCGAAATCGGTACGGATACCGCGAAAGAGCAGATTTATAACCGCTTCACACTGACGCCGGAAGGGGATGAACCGCTTCCCGGTGCCGTTCACTTCCCGAATAACCCGGATATTTTTGATCTGACCGAAGCGCAGCAGCTGACTGCTGAAGAGCAGGTCGAAAAATGGGTGGATGGCAGGAAAAAAATACTGTGGGACAGCAAAAAGCGACGCAATGAGGCACTCGACTGCTTCGTTTATGCGCTGGCGGCGCTGCGCATCAGTATTTCCCGCTGGCAGCTGGATCTCAGTGCGCTGCTGGCGAGCCTGCAGGAAGAGGATGGTGCAGCAACCAACAAGAAAACACTGGCAGATTACGCCCGTGCCTTATCCGGAGAGGATGAATGACGCGACAGGAAGAACTTGCCGCTGCCCGTGCGGCACTGCATGACCTGATGACAGGTAAACGGGTGGCAACAGTACAGAAAGACGGACGAAGGGTGGAGTTTACGGCCACTTCCGTGTCTGACCTGAAAAAATATATTGCAGAGCTGGAAGTGCAGACCGGCATGACACAGCGACGCAGGGGACCTGCAGGATTTTATGTATGAAAACGCCCACCATTCCCACCCTTCTGGGGCCGGACGGCATGACATCGCTGCGCGAATATGCCGGTTATCACGGCGGTGGCAGCGGAGCGT |
| seqCopy A-G mutation (Fig. 2d) | CGCTCCGCTACGAAATGCGCGTATGGGGATGGGGGCCGGGTGAGGAAAGCTGGCTGATTGACAAGCAGATTATTATGGGCCGCCACGACGATGAACAGACGCTGCTGCGTGTGGATGAGGCCATCAATACGACCTATACCCGCCGGAATGGTGCAGAAATGTCGATATCCCGTATCTAATGGGATACTGGCGGGATTGACCCGACCATTGTGTATGAACGCTCGAAGCAACATGGGCTGTTCCGGGTGATCCCCATTAAAGGGGCATCCGTCTACGGAAAGCCGGTGGCCAGCATGCCACGTAAGCGAAACAACTACGGGGTTTACCTTACCGAAATCGGTACGGATACCGCGAAAGAGCAGATTTATAACCGCTTCACACTGACGCCGGAAGGGGATGAACCGAATCCCGGTTTGGTTCACTTCCCGAATAACCCGGATATTCCTGATCTGACCGAAGCGCAGCAGCTGACTAATGAAGAGCAGGTCGAGCAATGGAAGGATGGCAGGAGGAGTATACTGAATGACAGCAGCAAGCGACGCAATGAGAAACTCGACTGCTTCGTTTATGCGCTGGCGGCGCTGCGCATCAGTATTTCCCGCTGGCAGCTGGATCTCAGTGCGCTGCTGGCGAGCCTGCAGGAAGAGGATGAAGCAGCAACCAACAAGAGTACACTGGCAGATTACGCCCGTGCCTTATCCGGAGAGGATGAATGACGCGACAGGAAGAACTTGCCGCTGCCCGTGCGGCACTGCATGACCTGATGACAGGTAAACGGGTGGCAACAGTACAGAAAGACGGACGAAGGGTGGAGTTTACGAACACTTCCGTGTCTGACCTGAGCAGGAATATATTGCAGAGCTGGAAGTGCAGACCGGCATGACACAGCGACGCAGGTTACCTGCAGGATGGTATGTATGACCACGCCCACCATTCCCACCCTTCTGGAAGGACGGCATGACATCGCTGCGCGAATATGCCGGTTATCACGGCGGTGGCAGCGGAGCGT |
| seqCopy A-T mutation (Fig. 2d) | CGCTCCGCTACGAAATGCGCGTATGGGGATGGGGGCCGGGTGAGGAAAGCTGGCTGATTGACCGGCAGATTATTATGGGCCGCCACGACGATGAACAGACGCTGCTGCGTGTGGATGAGGCCATCAATATATCCTATACCCGCCGGAATGGTGCAGAAATGTCGATATCCCGTATCTGCTGGGATACTGGCGGGATTGACCCGACCATTGTGTATGAACGCTCGATATATCATGGGCTGTTCCGGGTGATCCCCATTAAAGGGGCATCCGTCTACGGAAAGCCGGTGGCCAGCATGCCACGTAAGCGAAACATATACGGGGTTTACCTTACCGAAATCGGTACGGATACCGCGAAAGAGCAGATTTATAACCGCTTCACACTGACGCCGGAAGGGGATGAACCGCTTCCCGGTGCCGTTCACTTCCCGAATAACCCGGATATATATGATCTGACCGAAGCGCAGCAGCTGACTGCTGAAGAGCAGGTCGATATATGGGTGGATGGCAGGATATATATACTGTGGGACAGCATATAGCGACGCAATGAGGCACTCGACTGCTTCGTTTATGCGCTGGCGGCGCTGCGCATCAGTATTTCCCGCTGGCAGCTGGATCTCAGTGCGCTGCTGGCGAGCCTGCAGGAAGAGGATGGTGCAGCAACCAACAAGATATCACTGGCAGATTACGCCCGTGCCTTATCCGGAGAGGATGAATGACGCGACAGGAAGAACTTGCCGCTGCCCGTGCGGCACTGCATGACCTGATGACAGGTAAACGGGTGGCAACAGTACAGAAAGACGGACGAAGGGTGGAGTTTACGGCCACTTCCGTGTCTGACCTGTATATATATATTGCAGAGCTGGAAGTGCAGACCGGCATGACACAGCGACGCAGGGGACCTGCAGGATATAATGTATGATATCGCCCACCATTCCCACCCTTCTGGGGCCGGACGGCATGACATCGCTGCGCGAATATGCCGGTTATCACGGCGGTGGCAGCGGAGCGT |
| SeqB-base shuffle (Fig. 2f) | CGCTTACTCTGACTCAACTGATCTTCAGATCTTCACTTCTCACTAGATGTTCTGAGTGAGAGACACAGAAGACAGACATGACACACAGAGAGAAGAAGATCTACACTGAGATGTTGAACTACTCACTACTCTTCTGTGTCAACTATCTAGTTGAAGATGTTCATCAGTTGATGTGTTCATGAGATACATACTTGTGAGATGTGATGTTGAAGAAGTAGAACAACATCAGTTCACACAATGTTCACACACTGACACTACACATGTGTAGATGTTCAAGTAGTTGTTCAACTTCTCTCTACAGATGTGTTGTTGACAGATCATCATGTCTGAACAACTAGTCTGAACATCAGAGACTACTTGACTAGAATCACAGAAGTAGACAGAGATAGTAGTGATGTCTGTAGTCTGTTGAACAAGAGTCACTACTAGTAGAGAGACTACTCACATCACAGTGAGAGACACTCTACTCAGAGTGATGACACACTACTGACACATCACATAGATCAGATGCGT |
| SeqB-AT-tracts shuffle (Fig. 2f) | CGCTTTACTCATATATGTGCACCTATATCAACTGATCTTCAGCATCTTATAAATCATATCTTTCACCAGCATATAGTTTCTGGAAATGTGAGCCAGTAAATTGAAGGCAGCATATCGCGGGGAAATGGCGACACGGATTTATGTTGCTCATACTCTTCCTTATTCGAAGAATACCAGGGTTAAATATATGTCTCATGAGCATATATATAGGATACGAATGGCAAATAGGAATAGGTTCCTAATGCGCACCCCCGGTGCCACCGTAAGCGTTAATATATAGCGCGGAATACAGCTCCCGTATATGCCGCGGCATATACCCGGACTATTTACGAGATAGGGTTGAGTGTTGTTCTTATATATCAGTTTGATATGAACAAGAGTCTATTAAACACGAACGTGTTAAATGACTCCAACGTCAAAGATATTTGGCGCCGTCTATCAGAATATTATTGGCGATGGATTTCCCACTACAATAAGTGAACCATCACCATATATATACCAAGATATAGTGCGT |
| curved250 (Fig. 3f) | CGCTACGTCAGAGAATTCTGGCGAATCCTCTGACCACCATCGGAAAACTCCTGCTTTAGCAAGATTTTCCCTGTATTGTACAGAATCAGGGGATAACGCAGGAAAGAACACGAATATCATGGTGGAAAATGGCCGCTTTTCTGGATTCATCCGGATTGCTGGCAGAAACCCCCGGTATGACCGTGAAAACGGCCCGCTCTCGCCAGTTAATCCGGAGAGTCAGCGATGTTCCTGAGATGATGCGGAAGGTTACCTGGATTTTTTCAAAGGCAGCGT |
| TSS-rrsB (Fig. 4d) | CGCTTGTTACAAGTGCTGCCAGAGGGAACCCGGCTGGTGGATTCTGGCGCAGCGATTGCTCGCCGAACGGCCTGGTTGTTAGAACATGAAGCCCCGGATGCAAAATCTGCCGATGCGAATATTGCCTTTTGTATGGCAATGACGCCAGGAGCTGAACAATTATTGCCCGTTTTACAGCGTTACGGCTTCGAAACGCTCGAAAAACTGGCAGTTTTAGGCTGATTTGGTTGAATGTTGCGCGGTCAGAAAATTATTTTAAATTTCCTCTTGTCAGGCCGGAATAACTCCCTATAATGCGCCACCACTGACACGGAACAACGGCAAACACGCCGCCGGGTCAGCGGGGTTCTCCTGAGAACTCCGGCAGAGAAAGCAAAAATAAATGCTTGACTCTGTAGCGGGAAGCGT |
| TSS-polA (Fig. 4d) | CGCTCAGAAAACGACCCAAATAACGGATGATCCTTAAGGAGAAAAATAATTCATATCTATCCACATTAGAAAAAATCCCATTATCTCAATTATTAGGGATGGATTTATTTTTAACTGCATGAAAAACAAAGACAAACATCATGCTGTAAAAAGCATGATAATAAATTAAAAGCGATGTAAATAATTTATGCACAAAGTTATCCACATGACGATTTGCGAGCGATCCAGAAGATCTACAAAAGATTTTCACGAAAAGCGGTGAAAAACTCATGTTTTCATCCTGTCTGTGGCATCCTTTACCCATAATCTGATAAACAGGCACGGACATTATGGTTCAGATCCCCCAAAATCCACTTATCCTTGTAGATGGTTCATCTTATCTTTATCGCGCATATCACGCGTTTGCGT |
| AT-0.25 kb  (Fig. 2-Figure supplement 1) | CGCTATTCTTCGGTTAACGAAGTCTGTAATTGATTTGTTAAGTTTTTCTTTTGAATATACGCATTAATTTTAGATTGAATTTGCTGAAGCTCTTCTTGATCAGCTTCTCGAATGGTTTGCTCCTTTTGTTCGCTCGTTAACGATTGTGAAGAAGCAGGAGACTGATTAGGGGCTGTCTCCACTTCATTTGTTTTTTTCGTACTTTTAGCTGCAGGGGTATTTGAAGGCTGAACCGCTTCTTCAGATTCCTGCAGACGCGT |
| AT-0.5 kb  (Fig. 2-Figure supplement 1) | CGCTATTCTTCGGTTAACGAAGTCTGTAATTGATTTGTTAAGTTTTTCTTTTGAATATACGCATTAATTTTAGATTGAATTTGCTGAAGCTCTTCTTGATCAGCTTCTCGAATGGTTTGCTCCTTTTGTTCGCTCGTTAACGATTGTGAAGAAGCAGGAGACTGATTAGGGGCTGTCTCCACTTCATTTGTTTTTTTCGTACTTTTAGCTGCAGGGGTATTTGAAGGCTGAACCGCTTCTTCAGATTCCTGCAGAGATTGAAATTCCATCACGCCCGTTCCTCCAACGAACGCTTCATTAAATGCGCGAGAAAGGTTCTTAAATTTCTGCGCATCCACTGAGCTCATTGCAAACAATACAATGAATAAAGCAAGTAAAAGTGTAAGCAAATCTGAGTAAGGAAGCAGCCAGCTTTCGTCAACATGGTCCTCTTCATGCTTTCGTTTTCTGCGTCTACTCATTTATGCCCACTTCACTTTCTTGAAGAAGCTTTTTACGTTCCGCTCGCGT |
| AT- 1kb  (Fig. 2-Figure supplement 1) | CGCTATTCTTCGGTTAACGAAGTCTGTAATTGATTTGTTAAGTTTTTCTTTTGAATATACGCATTAATTTTAGATTGAATTTGCTGAAGCTCTTCTTGATCAGCTTCTCGAATGGTTTGCTCCTTTTGTTCGCTCGTTAACGATTGTGAAGAAGCAGGAGACTGATTAGGGGCTGTCTCCACTTCATTTGTTTTTTTCGTACTTTTAGCTGCAGGGGTATTTGAAGGCTGAACCGCTTCTTCAGATTCCTGCAGAGATTGAAATTCCATCACGCCCGTTCCTCCAACGAACGCTTCATTAAATGCGCGAGAAAGGTTCTTAAATTTCTGCGCATCCACTGAGCTCATTGCAAACAATACAATGAATAAAGCAAGTAAAAGTGTAAGCAAATCTGAGTAAGGAAGCAGCCAGCTTTCGTCAACATGGTCCTCTTCATGCTTTCGTTTTCTGCGTCTACTCATTTATGCCCACTTCACTTTCTTGAAGAAGCTTTTTACGTTCCGCTGTTGGCAAATAAGAAGCCAGCTTTTGCTCAATTACTTTTGGTGTTTCTCCTTCTAAAAGTGAAAGCACTCCTTCGATCATCATATACTTTACCTTTACTTCATGTTTCGATTTACGCTTTAGTTTATTTGCAAACGGATGCCATAGTACATACCCAGTAAAAATACCAAGAAGCGTAGCAACAAACGCCGCGCTGATCGCATGTCCTAGCGTATCTGTATCTTCCATGTTCCCAAGCGCAGCAATTAACCCTATAACAGCTCCAAGTACACCCAGAGTTGGAGCATATGTACCTGCTAAAGCGAAAATACTTGCACCCGTTTGATGTCTTTCTTCCATAGCATCAATTTCTTCAGACAACACGTCTCGTATATAATCCGCACTTTGACCATCAATAGCTAAATTCAAACCATTTTTTAAGAAAGGGTCATCTACATCAATAATTTGGGCTTCAAGTGATAGTAACCCTTCTTTTCGAACAACTTGTCCCCATTCAGAAAACGCGT |
| AT- 3 kb  (Fig. 2-Figure supplement 1) | CGCTATTCTTCGGTTAACGAAGTCTGTAATTGATTTGTTAAGTTTTTCTTTTGAATATACGCATTAATTTTAGATTGAATTTGCTGAAGCTCTTCTTGATCAGCTTCTCGAATGGTTTGCTCCTTTTGTTCGCTCGTTAACGATTGTGAAGAAGCAGGAGACTGATTAGGGGCTGTCTCCACTTCATTTGTTTTTTTCGTACTTTTAGCTGCAGGGGTATTTGAAGGCTGAACCGCTTCTTCAGATTCCTGCAGAGATTGAAATTCCATCACGCCCGTTCCTCCAACGAACGCTTCATTAAATGCGCGAGAAAGGTTCTTAAATTTCTGCGCATCCACTGAGCTCATTGCAAACAATACAATGAATAAAGCAAGTAAAAGTGTAAGCAAATCTGAGTAAGGAAGCAGCCAGCTTTCGTCAACATGGTCCTCTTCATGCTTTCGTTTTCTGCGTCTACTCATTTATGCCCACTTCACTTTCTTGAAGAAGCTTTTTACGTTCCGCTGTTGGCAAATAAGAAGCCAGCTTTTGCTCAATTACTTTTGGTGTTTCTCCTTCTAAAAGTGAAAGCACTCCTTCGATCATCATATACTTTACCTTTACTTCATGTTTCGATTTACGCTTTAGTTTATTTGCAAACGGATGCCATAGTACATACCCAGTAAAAATACCAAGAAGCGTAGCAACAAACGCCGCGCTGATCGCATGTCCTAGCGTATCTGTATCTTCCATGTTCCCAAGCGCAGCAATTAACCCTATAACAGCTCCAAGTACACCCAGAGTTGGAGCATATGTACCTGCTAAAGCGAAAATACTTGCACCCGTTTGATGTCTTTCTTCCATAGCATCAATTTCTTCAGACAACACGTCTCGTATATAATCCGCACTTTGACCATCAATAGCTAAATTCAAACCATTTTTTAAGAAAGGGTCATCTACATCAATAATTTGGGCTTCAAGTGATAGTAACCCTTCTTTTCGAACAACTTGTCCCCATTCAGAAAACATGGATACTAAATCAACGGGCTGCAGCATTTTTTGTTCTTTAAACAGGACACCAAACAGTTTTGGGACTCTTTTGATTTCATTTGAAGGAAATGCAATCACTACTGCTCCGACGGTTCCGACAATAATAATTAAAATAGCAGCCGGATTTCCTAACACAGATGGATTAACCCCTTTGAAAAACATACCTACAATAAGAGAGGCTATTCCTAAAATCAGTCCAATAAAAGATGTTTTATCCATATGTAATTCTCCTATCCTTAACTTCCTGTTCTTTCTTTTTGTGTATTTTATCCTCATCTTTTATTTCGACTATTTCATAGAATTTTTAAGCCTTTTATGAATCATATTTACAATTACAGCTTTTCTCTATTATAAAAACACTTTTTTCAACTTTTCATACAGAATATCTGAAGGTCCAGCTATAATCGCTACAATTTGATTTTCCACATAAATAGCAGCCGTCATCTTATTATCAATATCAGCTAAAATGGCAAATCCGACGTTAATGTTCAACTTTATTCACCTCTGTCACGTTCTTGTTTATTCAACATATAAAAAGAACCGTCTTACCATTTATTTTTCCGTGTCTTATTGACATTTGTAAATTTTATATTAAGATTATGTAAATGAGTTGACAAAATGGAGGTGATATCATGCTACTAGAAAAATACTGTAAAGACACTGATTTATTGATTATCCAGTTTACAATCGAACTAACAAAAGACATTCACGCTAAAATCTCCGCACGTACTTTATTTTATGAAGAACAGGTGATACGTTATGCTGAAAAAAGAATACGTTCTTTCTTACATCCTCTTTCCCTTAAACATACGCTAAAATTTGTCTATCAATCTGAAATACTACAAACCATTCTATTCAAATTAAAACCAACTTTTGAGCAGCAGCATGTATTGCGCTGTATTTCATCTTAAAAAGGAGTTCCCTTTAGCGGAAACTCCTTTTTGATTTGATTCTACGATATAGACGATATGCTTTTCTGATAACGTTTAACAGCGGGTACGGCAAGCGGATATGGTACTTATCAATAAACGGATAAAAGGTGTAATAAGCTTTTACAAGGTCTGTCCATTTTTTATCAAAACCTTCTTTATAAAAGTCAGAGATATCCACCACATACCCTCTTCCATCTTTCATCATGACATTTTTACCATGCACATCATATGGGTTTAAACCCTGGCTTCTTGCATAGTCCAAAGCTGCGTTCACATCTTTTATGACTTGTTCTGGAATCTTTATTCCCCGCTGGACGGCATCATACAAGGTAACGCCTGTAAGTCTTTTTAAAATCAAGTACGTCTTTCCCTCATGAAAAAGTTGAGAATAAGCTGGGTGAACGCCCAGCTTTCTATACACTTGCGCTTCTTTCTTTACACCATAAATTTCTCTTCCGTATACTTTCACGACAAATTCAGGATAATTTTCGTGCGTAAACACACCGGCATAGTTTCCTTTACCAATAAGTACCCACTCTTTTGTTTTGTTCGTAACTTCAACTGGATCATAGTCACTTTCACTTTGAATCGTAACTTGCGTTAGTAATGATGTTTCAACTAGCGACACCAACTGTTTAATTGTTTTATCCATAGTCCCCTCTAAAAATCCTTCAGTAATCTCTATCAAATATTACCCTATGATAAATCTCAATGCAGGATGTGTCAATAAATTGACAGCCTGATATAAAGAGGGAAAGTATTCCCGTTCATTCAAGACTGCGCGTGAACTTTGTGAACATTCACCTTCAGTTCTTCTTTCATATTCAGCATTACGCCCGCCATCGGAGCAATAGATTCGGGCTTAGTCAGCTGAAATAAAAAGCGCTTTGTCATTTCTGACAGAAAAATGGTGACTATTTCAGTGATATAGTCCATGCCCCTTTCCGCTTGATGCTCAATCCCAAGTGGCATATACATATAGCTTGGGACATCAATGGACCCTCCTTTTTCAAAACGGTCAGGTAAAAATTCACTTGGCTCTAACGCGT |
| AT- 3.9 kb  (Fig. 2-Figure supplement 1) | CGCTATTCTTCGGTTAACGAAGTCTGTAATTGATTTGTTAAGTTTTTCTTTTGAATATACGCATTAATTTTAGATTGAATTTGCTGAAGCTCTTCTTGATCAGCTTCTCGAATGGTTTGCTCCTTTTGTTCGCTCGTTAACGATTGTGAAGAAGCAGGAGACTGATTAGGGGCTGTCTCCACTTCATTTGTTTTTTTCGTACTTTTAGCTGCAGGGGTATTTGAAGGCTGAACCGCTTCTTCAGATTCCTGCAGAGATTGAAATTCCATCACGCCCGTTCCTCCAACGAACGCTTCATTAAATGCGCGAGAAAGGTTCTTAAATTTCTGCGCATCCACTGAGCTCATTGCAAACAATACAATGAATAAAGCAAGTAAAAGTGTAAGCAAATCTGAGTAAGGAAGCAGCCAGCTTTCGTCAACATGGTCCTCTTCATGCTTTCGTTTTCTGCGTCTACTCATTTATGCCCACTTCACTTTCTTGAAGAAGCTTTTTACGTTCCGCTGTTGGCAAATAAGAAGCCAGCTTTTGCTCAATTACTTTTGGTGTTTCTCCTTCTAAAAGTGAAAGCACTCCTTCGATCATCATATACTTTACCTTTACTTCATGTTTCGATTTACGCTTTAGTTTATTTGCAAACGGATGCCATAGTACATACCCAGTAAAAATACCAAGAAGCGTAGCAACAAACGCCGCGCTGATCGCATGTCCTAGCGTATCTGTATCTTCCATGTTCCCAAGCGCAGCAATTAACCCTATAACAGCTCCAAGTACACCCAGAGTTGGAGCATATGTACCTGCTAAAGCGAAAATACTTGCACCCGTTTGATGTCTTTCTTCCATAGCATCAATTTCTTCAGACAACACGTCTCGTATATAATCCGCACTTTGACCATCAATAGCTAAATTCAAACCATTTTTTAAGAAAGGGTCATCTACATCAATAATTTGGGCTTCAAGTGATAGTAACCCTTCTTTTCGAACAACTTGTCCCCATTCAGAAAACATGGATACTAAATCAACGGGCTGCAGCATTTTTTGTTCTTTAAACAGGACACCAAACAGTTTTGGGACTCTTTTGATTTCATTTGAAGGAAATGCAATCACTACTGCTCCGACGGTTCCGACAATAATAATTAAAATAGCAGCCGGATTTCCTAACACAGATGGATTAACCCCTTTGAAAAACATACCTACAATAAGAGAGGCTATTCCTAAAATCAGTCCAATAAAAGATGTTTTATCCATATGTAATTCTCCTATCCTTAACTTCCTGTTCTTTCTTTTTGTGTATTTTATCCTCATCTTTTATTTCGACTATTTCATAGAATTTTTAAGCCTTTTATGAATCATATTTACAATTACAGCTTTTCTCTATTATAAAAACACTTTTTTCAACTTTTCATACAGAATATCTGAAGGTCCAGCTATAATCGCTACAATTTGATTTTCCACATAAATAGCAGCCGTCATCTTATTATCAATATCAGCTAAAATGGCAAATCCGACGTTAATGTTCAACTTTATTCACCTCTGTCACGTTCTTGTTTATTCAACATATAAAAAGAACCGTCTTACCATTTATTTTTCCGTGTCTTATTGACATTTGTAAATTTTATATTAAGATTATGTAAATGAGTTGACAAAATGGAGGTGATATCATGCTACTAGAAAAATACTGTAAAGACACTGATTTATTGATTATCCAGTTTACAATCGAACTAACAAAAGACATTCACGCTAAAATCTCCGCACGTACTTTATTTTATGAAGAACAGGTGATACGTTATGCTGAAAAAAGAATACGTTCTTTCTTACATCCTCTTTCCCTTAAACATACGCTAAAATTTGTCTATCAATCTGAAATACTACAAACCATTCTATTCAAATTAAAACCAACTTTTGAGCAGCAGCATGTATTGCGCTGTATTTCATCTTAAAAAGGAGTTCCCTTTAGCGGAAACTCCTTTTTGATTTGATTCTACGATATAGACGATATGCTTTTCTGATAACGTTTAACAGCGGGTACGGCAAGCGGATATGGTACTTATCAATAAACGGATAAAAGGTGTAATAAGCTTTTACAAGGTCTGTCCATTTTTTATCAAAACCTTCTTTATAAAAGTCAGAGATATCCACCACATACCCTCTTCCATCTTTCATCATGACATTTTTACCATGCACATCATATGGGTTTAAACCCTGGCTTCTTGCATAGTCCAAAGCTGCGTTCACATCTTTTATGACTTGTTCTGGAATCTTTATTCCCCGCTGGACGGCATCATACAAGGTAACGCCTGTAAGTCTTTTTAAAATCAAGTACGTCTTTCCCTCATGAAAAAGTTGAGAATAAGCTGGGTGAACGCCCAGCTTTCTATACACTTGCGCTTCTTTCTTTACACCATAAATTTCTCTTCCGTATACTTTCACGACAAATTCAGGATAATTTTCGTGCGTAAACACACCGGCATAGTTTCCTTTACCAATAAGTACCCACTCTTTTGTTTTGTTCGTAACTTCAACTGGATCATAGTCACTTTCACTTTGAATCGTAACTTGCGTTAGTAATGATGTTTCAACTAGCGACACCAACTGTTTAATTGTTTTATCCATAGTCCCCTCTAAAAATCCTTCAGTAATCTCTATCAAATATTACCCTATGATAAATCTCAATGCAGGATGTGTCAATAAATTGACAGCCTGATATAAAGAGGGAAAGTATTCCCGTTCATTCAAGACTGCGCGTGAACTTTGTGAACATTCACCTTCAGTTCTTCTTTCATATTCAGCATTACGCCCGCCATCGGAGCAATAGATTCGGGCTTAGTCAGCTGAAATAAAAAGCGCTTTGTCATTTCTGACAGAAAAATGGTGACTATTTCAGTGATATAGTCCATGCCCCTTTCCGCTTGATGCTCAATCCCAAGTGGCATATACATATAGCTTGGGACATCAATGGACCCTCCTTTTTCAAAACGGTCAGGTAAAAATTCACTTGGCTCTAAAAAATAATCTTCATGGCGATGCATCATATAAGAGCTAATTAACATAATCTCCCCTTTTTTAATATTGTAGCCATCTATTTGTATATCTTCACGTGCTTGACGCCCAAATAGCCAAAGAGGCGGATACAACCTCATGCTTTCAGCAATTATTTTGCGCATATAGGTTAGATTTTTGGTTGAAAGTGATTCGCCGCTAGCATAGGCTTGAATTTCTTTATGCAGCTGAAGGTGCTCCCTAGTATTTTGAGACAGTAAATGAATAGACCAGCTGCATACGTGCGTAATCATTTCATACATCGAAAGGAATATAGAATTTAGCTGTTCGTATATTTCTCTTTCATCGCTGTCTTCTCCGTAAGAATTCAATATATACTGCAGTAAATCATTTCCTCCGGTTTTGTTTTGAATACGCATTTGAACGCGTTCAAAAAGGAGTTGTTCTAGCTGCTCATCTGAATCAGGCTGGTGCAAAGGCAGACGAATATAAATTTTGCCTAACTTTTCTTTTTTTCTCATAAGCGCTTGAACATAGTGAATTTTATCTTTCTCCTCAATCGAAATGCCAAATACAAGTTGAAGGAGTACAGCTACGACTATCTGTCTTATGTCTTTTACTATCGTTCGAAGCTGCCCTTCTTCCCACGTTTCCGTATGCTTTTCAATTATTTTTGCGATTGCTTCCTTATTATAAGTTAGATGTTGTTTTAGTTGTGAGGGTTGAATGGGGCTCATATATAATGCTTCATCTGTCCATAACATTTCTTCGCCTAGCAGCGTTTTAAACATATGCGTTAGTTTAATTTTTTGAAACGCCTTGCTGTTTGTAATGACTACATCTTTAATAAGTTGAGCGCGT |
| Flat500  (Fig. 3f) | TCACCAAGACGTACACTGAGCATTCGGGGAGCCGGTAGCGCGGACGCGGATTCGTTGCAGTGAAGCGGCACCGCCGGGTTCCCTCGTCGTGGTCTGCCCGTGCATGCACCACCATACGCACGGCCCTTCTGGGTGGATACACGGATAATATAGTCTAGGGTCCAGCGTAATAGGCGCCCGACAACCACGCTGCAAGGAAGGTGGCAGGTACGCGATCCGATCATCGACGGTGTGAGCCCGCTTTCTGGATCTTCCGCGTGCACGGCCCCGAAATCGCCCAGCTCGCCGGGACGTTCGGCCTGAAGTCGGCCGGCTTTCTGGGTCTCGGGATCCAACACGTTGTCGCGTAGGCCCCCCATGGCCGTTTGCCGTCGACACAGGGAGGCGGTTGGTTCAGCCGCTACATTGCGGTAGCTCCGTGGGGGTGGAGGAGGTGCCATCTAATTACAGGAACCTGTGGGCTGCAGAGAATCCACTGAGTCCGGGCCGCGGGTGATGGCCCCTATGGCAGTGCCTGGCTGGTCGCCTCCGCACCCCACGGGTTCCGCGTAGGGGGTTAGCGGTGGACTGAGCGA |
| Curve75-1  (Fig. 3g) | CGAATCAGCCTATTTAGGCTATTTTTTCCACCATTTCTGGCGTTATTTCCGGTTTTTACTGAGATCTCTCCCACT |
| Curve75-2  (Fig. 3g) | GATGCTCACCGCATTTCCTGAAAATTCACGCTGTATCTTGAAAAATCGACGTTTTTTACGTGGTTTTCCGTCGAA |
| Flat75-1  (Fig. 3h) | AGGGGGGCGGCTATTGAAGGGGTCCCGTTGGTCTGCCCACAAAAGGCCCCCTGAGTCTTTCACTCAGTTTGTCCT |
| Flat75-2  (Fig. 3h) | ACAGGTCCCGGGTGTTCACGCATCCACAGACCCCTCGGAGGGAGCATTAGAGGCTGGGGCTCATCTGGGGGTCTG |
| GCcurve1  (Fig. 3i) | TCACCCTTCCGCGTGCACGGCCCCGAAATCGCCCAGCTCGCCGGGACGTTCGGCCTGAAGTCGGCCGGCTTTCTGGGTCTAAGACGTACACTGAGCATTCGGGGAGCCGGTAGCGCGGACGCGGATTCGTTGCAGTGAAGCGGCACCGCCCTTCCGCGTGCACGGCCCCGAAATCGCCCAGCTCGCCGGGACGTTCGGCCTGAAGTCGGCCGGCTTTCTGGGTCTGGGTTCCCTCGTCGTGGTCTGCCCGTGCATGCACCACCATACGCACGGCCCTTCTGGGTGGATACACGGACTTCCGCGTGCACGGCCCCGAAATCGCCCAGCTCGCCGGGACGTTCGGCCTGAAGTCGGCCGGCTTTCTGGGTCTTAATATAGTCTAGGGTCCAGCGTAATAGGCGCCCGACAACCACGCTGCAAGGAAGGTGGCAGGTACGCGACTTCCGCGTGCACGGCCCCGAAATCGCCCAGCTCGCCGGGACGTTCGGCCTGAAGTCGGCCGGCTTTCTGGGTCTTCCGATCATCGACGGTGTGAGCCCGCTTTCTGGATCGGGATCCAACACGTTGTCGCGTAGGCCCCCCATGCTTCCGCGTGCACGGCCCCGAAATCGCCCAGCTCGCCGGGACGTTCGGCCTGAAGTCGGCCGGCTTTCTGGGTCTGCCGTTTGCCGTCGACACAGGGAGGCGGTTGGTTCAGCCGCTACATTGCGGTAGCTCCGTGGGGGTGGAGCTTCCGCGTGCACGGCCCCGAAATCGCCCAGCTCGCCGGGACGTTCGGCCTGAAGTCGGCCGGCTTTCTGGGTCTGAGGTGCCATCTAATTACAGGAACCTGTGGGCTGCAGAGAATCCACTGAGTCCGGGCCGCGGGTGATGGCCTTCCGCGTGCACGGCCCCGAAATCGCCCAGCTCGCCGGGACGTTCGGCCTGAAGTCGGCCGGCTTTCTGGGTCTCCCTATGGCAGTGCCTGGCTGGTCGCCTCCGCACCCCACGGGTTCCGCGTAGGGGGTTAGCGGTGGACTGCTTCCGCGTGCACGGCCCCGAAATCGCCCAGCTCGCCGGGACGTTCGGCCTGAAGTCGGCCGGCTTTCTGGGTCTAGCGA |
| GCcurve2  (Fig. 3i) | TCACCGTACGAGGCGGGCGCGAGCTCGTGGTTGTTCTCCACGAACTCCCCGTAGTTCACGAGGTATTCGCCGACGTCGAGAAGACGTACACTGAGCATTCGGGGAGCCGGTAGCGCGGACGCGGATTCGTTGCAGTGAAGCGGCACCGCCGTACGAGGCGGGCGCGAGCTCGTGGTTGTTCTCCACGAACTCCCCGTAGTTCACGAGGTATTCGCCGACGTCGAGGGGTTCCCTCGTCGTGGTCTGCCCGTGCATGCACCACCATACGCACGGCCCTTCTGGGTGGATACACGGAGTACGAGGCGGGCGCGAGCTCGTGGTTGTTCTCCACGAACTCCCCGTAGTTCACGAGGTATTCGCCGACGTCGAGTAATATAGTCTAGGGTCCAGCGTAATAGGCGCCCGACAACCACGCTGCAAGGAAGGTGGCAGGTACGCGAGTACGAGGCGGGCGCGAGCTCGTGGTTGTTCTCCACGAACTCCCCGTAGTTCACGAGGTATTCGCCGACGTCGAGTCCGATCATCGACGGTGTGAGCCCGCTTTCTGGATCGGGATCCAACACGTTGTCGCGTAGGCCCCCCATGGTACGAGGCGGGCGCGAGCTCGTGGTTGTTCTCCACGAACTCCCCGTAGTTCACGAGGTATTCGCCGACGTCGAGGCCGTTTGCCGTCGACACAGGGAGGCGGTTGGTTCAGCCGCTACATTGCGGTAGCTCCGTGGGGGTGGAGGTACGAGGCGGGCGCGAGCTCGTGGTTGTTCTCCACGAACTCCCCGTAGTTCACGAGGTATTCGCCGACGTCGAGGAGGTGCCATCTAATTACAGGAACCTGTGGGCTGCAGAGAATCCACTGAGTCCGGGCCGCGGGTGATGGCGTACGAGGCGGGCGCGAGCTCGTGGTTGTTCTCCACGAACTCCCCGTAGTTCACGAGGTATTCGCCGACGTCGAGCCCTATGGCAGTGCCTGGCTGGTCGCCTCCGCACCCCACGGGTTCCGCGTAGGGGGTTAGCGGTGGACTGGTACGAGGCGGGCGCGAGCTCGTGGTTGTTCTCCACGAACTCCCCGTAGTTCACGAGGTATTCGCCGACGTCGAGAGCGA |
