# Supplementary material for: DNA sequence encodes the position of DNA supercoils
Source: eLife. 2018 Dec 7;7:e36557. doi: 10.7554/eLife.36557 (PMC6301789; doi:10.7554/eLife.36557)
Supplement: Supplementary file 3. [file elife-36557-supp3.docx]

**Supplementary File 3**

| **Template 1** | | | |
| --- | --- | --- | --- |
| **DNA fragment** | **Primers** | **Template** | **PCR or hybridization** |
| Biotin-handle | GACCGAGATAGGGTTGAGTG | pBlueScriptIISK + | PCR (taq), bio-11-dUTP |
|  | TTTTTTTTTTGGTCTCTCCAGCTGGCGTTACCCAACTTAATCGCC |  |  |
| 11.2 kb fragment | TTTTTTGGTCTCACTGGCAGGAACAGGGAATGC | Unmethylated Lambda DNA | PCR (phusion) |
|  | TTTTTTGGTCTCTACGCGCGTGCCCATGTTCTCTTTCAG |  |  |
| 8.4 kb fragment | TTTTTTGGTCTCTGCGTATAAGAAAGCAGACGACATCTGG | Unmethylated Lambda DNA | PCR (phusion) |
|  | TTTTTTGGTCTCCATACACGGTGATGGTCCCGG |  |  |
| Biotin-Cy5 handle | GACCGAGATAGGGTTGAGTG | pBlueScriptIISK + | PCR(Gotaq), Bio-11-dUTP+ Aminoallyl-dUTP-Cy5 |
|  | TTTTTTTTTTGGTCTCTGTATCTGGCGTTACCCAACTTAATCGCC |  |  |
