# Supplementary material for: DNA sequence encodes the position of DNA supercoils
Source: eLife. 2018 Dec 7;7:e36557. doi: 10.7554/eLife.36557 (PMC6301789; doi:10.7554/eLife.36557)
Supplement: Supplementary file 4. [file elife-36557-supp4.docx]

**Supplementary File 4**

| **Template 2** | | | | |
| --- | --- | --- | --- | --- |
| **DNA fragment** | **Primers** | **Template** | **PCR or hybridization** | **Restriction enzyme** |
| Biotin-handle | GACCGAGATAGGGTTGAGTG | pBlueScriptIISK + | PCR (taq), bio-11-dUTP | XhoI |
|  | CAGGGTCGGAACAGGAGAGC |  |  |  |
| Biotin-Cy5 handle | GACCGAGATAGGGTTGAGTG | pBlueScriptIISK + | PCR(Gotaq), Bio-11-dUTP+ Aminoallyl-dUTP-Cy5 | NotI-HF |
|  | CAGGGTCGGAACAGGAGAGC |  |  |  |
| pSuperCos-λ1,2 | X | X | X | NotI-HF + XhoI |
